# Recent Advances in Degradable Hybrids of Biomolecules and NGs for Targeted Delivery

**DOI:** 10.3390/molecules24101873

**Published:** 2019-05-15

**Authors:** Iwona Stanislawska, Wioletta Liwinska, Marek Lyp, Zbigniew Stojek, Ewelina Zabost

**Affiliations:** 1 Department of Nutrition, College of Rehabilitation, Kasprzaka 49, 01-234 Warsaw, Poland; iwona.stanislawska@wsr.edu.pl (I.S.); marek.lyp@wsr.edu.pl (M.L.); 2 Faculty of Chemistry, University of Warsaw, Pasteura 1, 02-093 Warsaw, Poland; wliwinska@chem.uw.edu.pl (W.L.); stojek@chem.uw.edu.pl (Z.S.)

**Keywords:** controlled release, targeted delivery, hybrid nanogels, biomolecule–hydrogel nanocomposites, drug delivery systems, smart materials, high-drug loading, on–off systems, remotely controlled release

## Abstract

Recently, the fast development of hybrid nanogels dedicated to various applications has been seen. In this context, nanogels incorporating biomolecules into their nanonetworks are promising innovative carriers that gain great potential in biomedical applications. Hybrid nanogels containing various types of biomolecules are exclusively designed for: improved and controlled release of drugs, targeted delivery, improvement of biocompatibility, and overcoming of immunological response and cell self-defense. This review provides recent advances in this rapidly developing field and concentrates on: (1) the key physical consequences of using hybrid nanogels and introduction of biomolecules; (2) the construction and functionalization of degradable hybrid nanogels; (3) the advantages of hybrid nanogels in controlled and targeted delivery; and (4) the analysis of the specificity of drug release mechanisms in hybrid nanogels. The limitations and future directions of hybrid nanogels in targeted specific- and real-time delivery are also discussed.

## 1. Introduction

Rapid progress in the design of hybrid nanogels (NGs) dedicated to various biomedical applications has recently been seen. Polymer-based NGs, incorporating biomolecules into their nanonetworks, are promising drug carriers. They gain a great potential in nanomedicine, production of pharmaceutics, biosensing, and biotechnology. In this context, hybrid NGs, as multifunctional structures that possess great ability to adapt to dedicated places, are called “the next generation drugs” [1].

The approaches to the design of polymeric hybrid NGs are exclusively directed towards development of controlled release- and targeted-delivery carriers. The hybrid NGs are promising in advanced therapies, where high specificity-, high efficacy-, and low adverse effects are desirable. Generally, an effective drug carrier should be capable of: (a) combining strong targeted delivery aspects with controlled drug redistribution in cancer tissue and (b) effectively overcoming drug resistance. The main challenge in the construction of the locally operating drug carriers is to keep the drug distribution possibly uniform in cancer tissues during the entire treatment cycle, and to avoid frequent injections of the chemotherapeutic drug. Furthermore, maintaining the minimum therapeutic concentration during the treatment time must be considered. Also, the effective carrier should be successfully metabolized and removed from the body. Finally, the single injection-based nanocarrier that possesses the ability for high-capacity drug loading and for prolonged, sustained and controlled drug release would be a great advantage [1,2].

The effective overcoming of drug resistance is obtained through different mechanisms; this phenomenon is often called multidrug resistance (MDR). Several processes stand behind MDR: faster detoxification and decreased uptake of drugs, increased intracellular nucleophiles levels, enhanced repair of drug-induced DNA damage, overexpression of drug transporters such as P-glycoprotein (P-gp), multidrug resistance-associated proteins (MRP1, MRP2), and breast cancer resistance protein (BCRP). Numerous mechanisms of drug resistance were briefly reported in Reference [3]. Nanocarriers, including hybrid NGs, have the potential to improve the drug therapeutic index, the ability for multifunctionality, to divert the ATP-binding cassette (ABC) transporter-mediated drug efflux mechanism, and to selectively target cancer cells or the cancer microenvironment. Unique combinations of hybrid polymeric NGs to overcome drug resistance mechanisms are directed at:

- degradable conjugate of anticancer drugs with P-gp protein substrates [4,5],
- modification of NGs with gene silencing P-gp expression substrates, e.g., with iRNA, monoclonal antibody, peptides, and its combination with anticancer drugs in co-delivery processes [6,7],
- degradable conjugate of various affinity ligands with cancer cells receptors, e.g., biotin, folic acid (FA) [8,9],
- dual drug conjugation/encapsulation strategies in the synthesis of hybrid NGs [10,11].

In general, typical NGs are flexible, deformable, and versatile nanotechnology platforms, comprised of highly hydrated, crosslinked hydrophilic polymers. They quickly respond to external stimuli. They are able to change their swelling, permeability, viscoelasticity, and hydrophobicity (or hydrophilicity) parameters. Nanogels, as drug delivery carriers, possess other advantages: they (a) reduce off-target effects, (b) are highl stable in body fluids, (c) extend drug circulation time, (d) deliver drugs to specific tissues, and (e) control drug release. The introduction of biomolecules into NGs provides an opportunity to: (a) moderate the physicochemical changes of parameters of NGs and respond to external stimuli, (b) improve storage capacity and drug release aspects, (c) introduce novel treatment methods for biomolecule-based height–weight therapeutics, (d) decrease drug toxicity and preserve the active form of the drug, (e) improve degradability and utilization of nanogel-based carriers, and (f) effectively overcome drug cellular resistance [12,13].

Another great advantage of biomolecule-modified NGs is the possibility of attaching them to electrode and conducting surfaces. Nanogels layers can be effectively used for conjugation and entrapment of peptides, proteins, and oligonucleotides. Several studies demonstrated that flexible and tunable NGs are effective platforms for the construction of wearable electronic devices, biosensors, and “system-on-chip” devices [14,15,16].

Although NGs are promising in terms of their application in the targeted therapies and in controlled delivery, they still have several limitations. Only a few NGs have been tested in clinical trials. There is a lack of large-scale investigations that deeply analyze the problem of NGs’ behavior under in vivo conditions. There is a need for deep evaluations of the biocompatibility, biodegradability, biodistribution, and elimination of NGs after in vivo administration.

In particular, the routes for combinations of biomolecules and NGs are focused on improvement and progress in the following aspects [17,18,19,20,21,22]:

- increase in efficiency of drug loading,
- improvement in controlled release and targeted delivery,
- extension of time of nanogel systemic circulation,
- ability to absorb large amounts of various liquids,
- control of flexibility and mechanical properties of NGs, including the tunability of their sizes,
- ability to simultaneously encapsulate several drugs,
- ability of the hybrids to be loaded with various types of drugs,
- improvement in nanogel stability in aqueous solutions and in the presence of serum,
- increase in lifetime of drug presence inside the nanogel,
- introduction of multi-stimuli responsiveness to the NGs,
- ability to degrade for effective removal with kidneys or liver,
- improvement in nanogel non-toxicity and biocompatibility,
- ability to respond in a controlled, switchable way to environmental changes (on–off systems),
- possibility of remote control for effective release and delivery.

**Figure 1:**
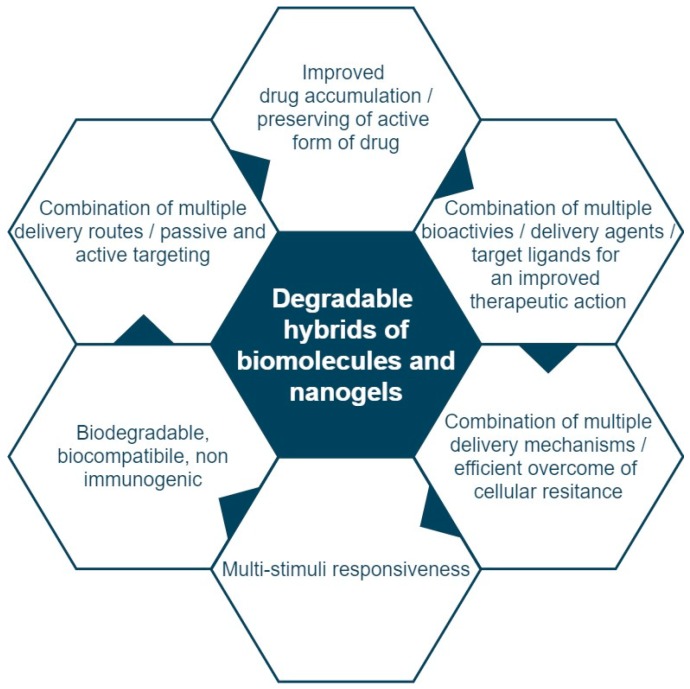
Major advantages of application of degradable hybrids of biomolecules and nanogels (NGs) as controlled drug-release and targeted delivery carriers.

An overview of major advantages of degradable nanogel hybrids containing biomolecules is presented in Figure 1. Our review provides an update on recent advances, and focuses on: (1) the changes in the key physical factors of hybrid NGs resulting from the introduction of biomolecules; (2) advances in synthesis and bioconjugation processes in degradable biomolecule/nanogel hybrids; (3) usefulness of biomolecules for self-organization of NGs and design of nanogel-based networks; (4) advantages of application of hybrid NGs for controlled and targeted delivery; and (5) analysis of the specificity of the release mechanism of drugs from hybrid NGs.

## 2. Design Criteria for Degradable Nanogel and Biomolecule Hybrids

Various types of macroscopic- and nanoparticle-based systems have been developed and applied for drug delivery, including hydrogels, polymer-based nanoparticles, micelles, liposomes, as well as inorganic particles. All kinds of delivery systems exhibit high levels of drug loading, drug protection, and the ability to control the rate of drug release and distribution. The nano-sized systems possess the unique following properties: administration by injection, overcoming biological barriers, and accumulation in sites of high vascular permeability. They have relatively large surfaces and can be modified by various affinity ligands on a rather large scale. The hydrogels, as macroscopic delivery systems, exhibit the ability to load large amounts of water; thus, they are highly biocompatible. They have been designed for engineering scaffolds and for local delivery of biomolecules and drugs. The NGs are very deformable, flexible, and viscoelastic; thus, they possess a very high ability to be effectively transported in body capillaries and to pass through cell membranes. It should be stressed, here, that effective cell internalization of NGs strongly depends on their size, surface chemistry, and surface charge. Due to their internal modification by crosslinking, NGs have the unique capability to control the degradation and the release of small compounds and biomolecule-based biotherapeutics. Furthermore, NGs are highly biocompatible due to their hydrophilic nature. This provides an opportunity to attach and load large amounts of hydrophilic biomolecules/drugs. The NGs can easily by modified by changing crosslinking density, size, and number of levels of NGs layers and surface modification. Finally, the NGs can be combined to large macroscopic surfaces (hydrogels) with controlled organization of the structure [23,24].

If biomolecules are selected as delivery biotherapeutics, it should be stressed that many of those height–weight agents possess their therapeutic actions inside the cells, e.g., in cytoplasm and in lysosomes. The delivery of biomolecules to the cell environment is challenging due to their inability to free pass through the cell membranes. Also, many of biomolecules can be quickly removed by renal filtering and scavenger cells in the liver, and be inactivated in the enzymatic-based processes. Effectively designed hybrid nanoparticles should be internalized to cells by cell uptake processes, e.g., endocytosis, phagocytosis, and their localization in endo/lysosomes. Also, the mechanism of endo/lysosome escape of internalized NGs should take place. An effective and rapid NG degradation inside the cells and the removal of the NGs constituents should take place [25,26].

In general, material selection and network fabrication in polymer-based NGs have a significant influence on the rate and mode of drug release and the type of delivery mechanism. Typical hybrid nanogel is defined as a nanogel complex composed of hydrophilic or amphiphilic polymer-based lattice and grafted/conjugated biomolecules. The introduction of biomolecules into the nanogel network results in the appearance of several new physical, biological, and transport properties, compared to unmodified NGs. These phenomena are discussed in the following subsections.

### 2.1. Physical and Biological Properties of Biomolecule/Nanogel Hybrids

#### 2.1.1. Morphology

**Figure 2:**
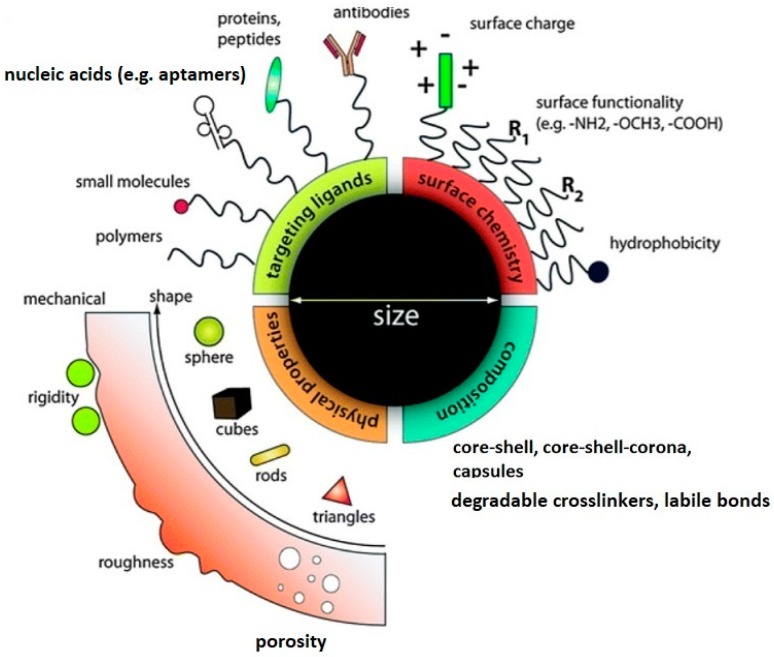
Design aspects of degradable biomolecule/nanogel hybrids for targeted delivery. Various types of biomolecules, e.g., proteins, nucleic acids, and antibodies, can be accumulated/grafted in NGs with specific routes of surface activation and moderation. Reprinted from Reference [30] with permission from the Royal Society of Chemistry.

Rigorous control of the size of each type of nanogel is critical from the point of biomedical applications. Taking into account the way of drug administration, the NGs can be easily applied by intravenously, compared to micro-sized gels. The NGs’ sizes allow them to move through the cell capillaries due to the enhanced permeability and retention (EPR) effect of diseased cells and to extravasate through the capillary endothelium to reach the target tissues [27,28]. Moreover, the size of an environmentally sensitive nanogel strongly determines the rate of the volume phase transition process which proceeds according to the diffusion-controlled mechanism [29]. The diameter of a typical hybrid nanogel amounts from 10 to 200 nm. The NGs are based on tridimensional porous cross-linked networks which results in relatively large surface areas and allows high drug-loading capacity and the possibility for multivalent biomolecule conjugations, see Figure 2 [30].

The most common synthesis ways proposed for hybrid biomolecule/nanogel hybrids stand with the ‘‘bottom-up’’ approach [31]. In this approach, the design process of NGs starts from a mixture of the monomer and a biomolecule and the obtained net is either physically or chemically (covalently) cross-linked. Another way is the assembling of hybrid NGs from polymer precursors and biomolecules [32]. In general, biomolecules can be introduced into the NGs by several methods [33,34,35]:

- by covalent bonding to the polymer-chains network during the polymerization process,
- by covalent bioconjugation after the polymerization process, that stands with grafting the biomolecules with an application of labile bonds onto the outer surface (mainly) of the NGs,
- by physical crosslinking/entrapment/self-assembly in nanogel nets during and after the polymerization process, inside and on the outer nanogel surface.

Compared to chemically crosslinked NGs by covalent bonds, those physically crosslinked and self-assembled under mild conditions possess greater ability to non-destructively introduce the biomolecules. Weak interactions, i.e., hydrogen bonding and hydrophobic and ionic interactions between the polymer chains, take part in this type of synthesis process. In the covalent crosslinking-based NGs, the conjugation/activation chemistry allows to design spatially organized hierarchical nanogel structures at the molecular level; in such a way, the biomimetic nanogel hybrids can be obtained [36].

As the physical assembly generates a higher risk of adverse effects due to the low stability of the NGs in body fluids, this process, when followed by the chemical conjugation, gives a significant opportunity for the construction of highly stable, biodegradable hybrid NGs made from biopolymers. Recent investigations show that there is an opportunity for the design of the hybrid NGs in a high-throughput manner [37].

The control of the chemical composition and of the balance between the amount of hydrophobic and hydrophilic moieties in the hybrid nanogel nets is another important aspect that influences the size of the nanogel hybrids. It is agreed, that nanogel hybrids should possess a hydrophilic shell/external layer for preserving their high biocompatibility [38]. On the other hand, the presence of hydrophilic biomolecules can increase the nanogel size due to the increased accumulation of water-based solutions. Thus, an important thing in the synthesis of hybrid NGs is to take control through a combination of the gel’s chemical composition and application of the physicochemical factors such as pH, temperature, and ionic strength. As well, the control over crosslinking density of the nanogel nets allows tuning of the nanogel size. In general, two main strategies exist in the design of nano-sized biocompatible hybrid NGs [39,40]:

- the design of specifically spatially organized structures of hybrid NGs, e.g., core-shells, for control of the water adsorption,
- the control of the hydrophilicity by changing the crosslinking density, e.g., by using of biomolecules as internal nanogel crosslinkers.

Another important aspect is to keep under control the process of aggregation and self-assembly of hybrid NGs. This is because the smaller nanogel sizes provide increased blood circulation and better cellular uptake [41,42]. Typically, the self-assembly process starts due to the existence of non-covalent weak interaction, e.g., electrostatic and hydrophobic. It is a diffusion process. It determines conformational and spatial structures of biomolecules in the hybrid NGs. The controlled self-assembly process of the hybrid NGs influences not only the aggregates sizes but also triggers the substantial drop in the nanogels’ sizes [43].

**Figure 3:**
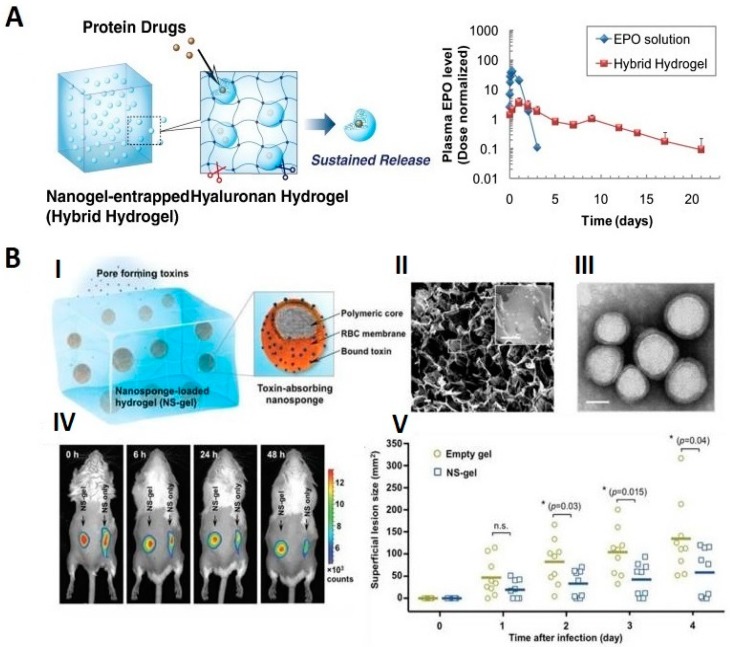
Example constructions of macroscopic hydrogels with entrapped biomolecule hybrid NGs for controlled release of drugs. (**A**) Hybrid macroscopic hydrogel constructed from protein-based/polymeric nanogel and hyaluronic-based (HA) hydrogel, and the sustained release of molecular chaperone-like active therapeutic proteins. Reprinted from Reference [47] with permission from the Elsevier. (**B**) Poly(lactic-co-glycolic acid), (PLGA) polymeric-multilayer nanosponge-loaded polyethylene glycol dimethacrylate (PEGDMA)-based hydrogel, (NS-gel) dedicated to local treatment of methicillin-resistant *Staphylococcus aureus* (MRSA) infection. (**I**) Scheme of NS-gel, with nanosponges loaded with a pore-forming bacterial protein toxins (PFTs) drug and (**II**) SEM image of NS-gel sample. The scale bar: 1 µm. (**III**) Transmission electron microscopy (TEM) image showing the spherical core-shell structure of nanosponges. The scale bar: 50 nm. (**IV**) Fluorescence images taken at different times present retention of nanosponges under mouse skin. (**V**) Quantification of fluorescence intensity as observed in (**IV**). Error bars represent standard deviation (*n* = 3). Reprinted from Reference [48] with permission from the John Wiley and Sons.

A typical single particle of the hybrid nanogel possesses a spherical-solid structure. It can form a core-shell object. The particles often possess irregular surfaces due to the grafting of various biomolecules onto the outer NGs surfaces. In recent times, a specific trend has been seen. This is the generation of micropatterns and organized macroscopic structures/scaffolds built from biomolecules and NGs; example constructions are presented in Figure 3. They are designed for the implantation and injection approaches. Specific environmental responsiveness of such scaffolds stands with the controlled release and targeted delivery of drugs. Especially, the trend of the application of the hydrogel and nanogel composites in the complex treatment of treatment-resistant diseases is seen. In fact, single nanogel or hydrogel-based therapy can be insufficient [44,45,46,47,48].

#### 2.1.2. Swelling/Shrinking—Volume Phase Transition

**Figure 4:**
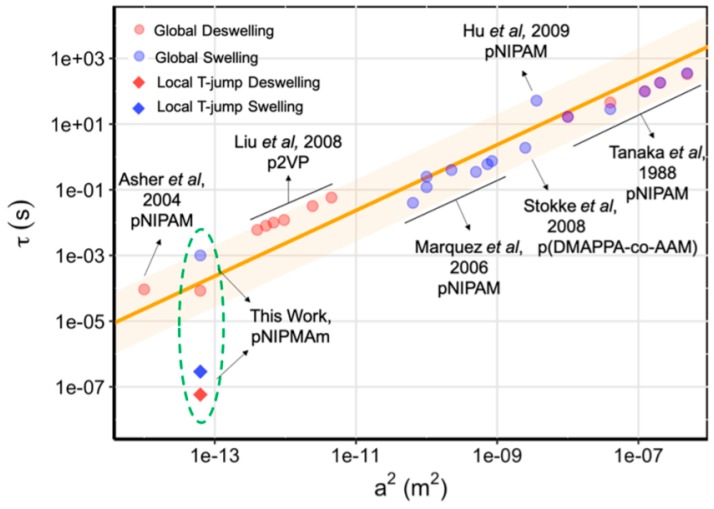
Swelling−deswelling kinetics (log–log correlation plot) of typical-size NGs observed using the Tanaka model by the correlation between the particle deswelling−swelling time constant, τ, and the square of the particle radius, a^2^. Reprinted with permission from Reference [49]. Copyright 2019 American Chemical Society.

The swelling behavior of NGs is the most important property related to enlargement of nanogel structures due to solvent accumulation in the void spaces between the polymer chains. As it was mentioned, the size of NGs, the duration time of the nanogel collapse effect, and the versatility of volume phase transition are strongly size-dependent, see Figure 4 [49]. In general, polymers and monomers usually applied for the design of hybrid NGs for biomedical application (e.g., poly(*N*-isopropylacrylamide, PNIPA) possess a volume phase transition temperature (VPTT) lower than that of the physiological use; it is in the range of 31–34 °C [50,51].

In general, the major abilities of many polymeric-based and crosslinked NGs are: (a) the high-level absorption of solvent, up to 95% and (b) the occurrence of the reversible volume phase transition effect. The explicitly high-absorption capacity of water-based solvents is related to the presence, in the nanogel nets, of additional hydrophilic groups, e.g., –OH, –CONH–, –CONH_2_–, and –SO_3_H. Strong hydrophilic lattices of the hydrogels possess properties of solids and fluids. In the macroscale, typical hydrogels behave as solids, where the polymeric network marks its shape and rheological properties. On the contrary, the NGs possess the properties of liquids because small-weight compounds can migrate and diffuse in the net, while high-weight compounds can be easily trapped in them. Correspondingly, the introduction of biomolecules into the nanogel network can change the absorption capacity of the NGs [48,51,52,53,54].

During the volume phase transition process, the swollen NGs that absorb high amounts of the solvent can minimize their volumes and reach the shrunken state. Then the solvent is removed from the nanogel tridimensional networks. The cause of this effect is two processes: (a) change in the balance between repulsive and attractive forces and (b) change in the osmotic pressure. The repulsive forces stimulate the swelling process of the NGs; mostly, these are electrostatic interactions between similarly charged groups. The attractive forces which are related to the NGs shrunken state are typically hydrophobic or Van der Vaals interactions [55].

The volume phase transition in the NGs can be triggered by both: physical and chemical factors, e.g., temperature, pH, light, pressure, electric field, and the presence of specific molecules. This property makes them stimuli-responsive [56]. In general, the NGs that possess the ability of environmental sensitiveness are called “smart” or “intelligent” NGs. Representative responsiveness of the degradable hybrid NGs is presented in Table 1. The introduction of multi-sensitiveness allowed the design of most advanced hybrid nanogel structures [57].

The most common hybrid NGs for targeted delivery are designed to be environmentally responsive, e.g., sensitive to temperature and/or pH. The chemical composition of the hybrid NGs (they exhibit usually different lower critical solution temperatures, LCSTs) will influence the temperature of the transition between the swollen and shrunken NG states.

**Figure 5:**
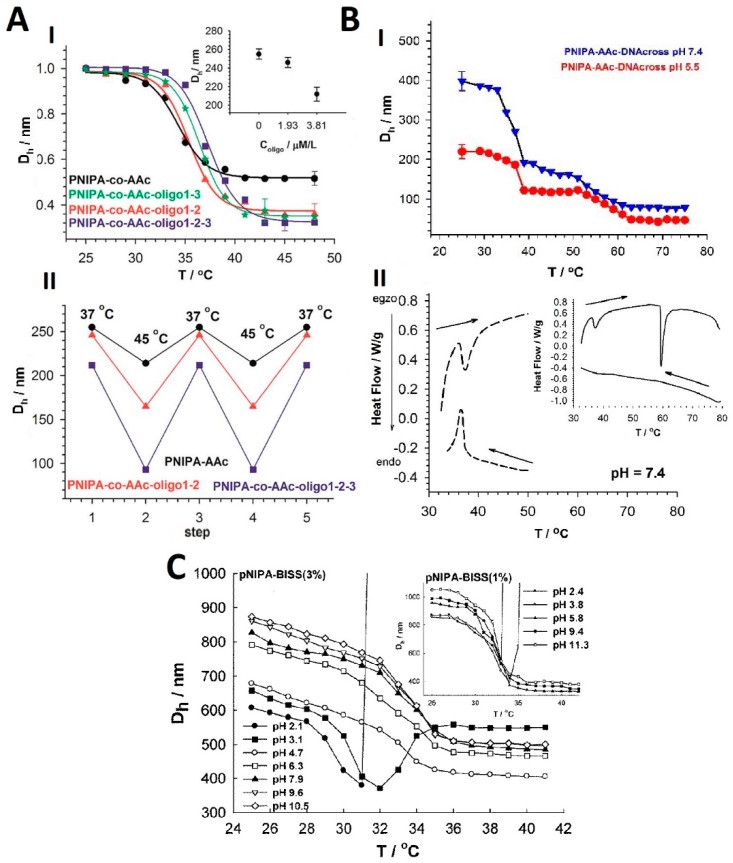
(**A**) The substantial movement of VPTT temperatures towards higher values after introducing of specific tri-segment structure of oligonucleotides to PNIPA-AAc based nondegradable NGs (PNIPA-co-AAc-oligo1-2-3 sample, I), significant reversibility of the size changes for hybrid oligonucleotide/PNIPA-AAc NGs contained tri-segment oligonucleotide structures in mild hyperthermia treatment (II), (**B**) the jumping VPTT changes recorded during degradation nanogel contained oligonucleotide-based crosslinkers (I), DSC plots recorded during degradation of oligonucleotide/PNIPA-AAc based nanogel hybrids (II), (**C**) Hydrodynamic diameter as function of temperature and pH for degradable, GSH responsive PNIPA-based microgel contained 3% of *N*,*N*-bisacryloylcystine (BISS) crosslinker. Reprinted from [50,51,76] with permission from the Royal Society of Chemistry, respectively.

Biomolecules accumulated/grafted/bioconjugated to the nanogel network can move the VPTT to higher temperatures [50,51,52]. Despite the promising features of biomolecule crosslinkers, only a few examples were presented in the literature for the design of thermoresponsive hybrid NGs, perhaps because of the difficulty of its conjugation with nanogel surfaces. Liwinska et al. [50,51] proposed several approaches for covalent binding of oligonucleotides as the crosslinkers in temperature- and pH-responsive hybrid NGs. The introduction of programmed specific tri-segment structures of oligonucleotides resulted in an increase in VPTT to 37 °C, see Figure 5A(I). Also, due to increasing the range of hydrodynamic diameter changes, the change in volume referred to the NGs phase transition was the largest for hybrid oligonucleotide-polymeric NGs containing specific tri-segment structures od oligonucleotides, see Figure 5A(II). The pH and temperature-sensitive degradable NGs containing oligonucleotide and disulphide (SS)-based crosslinkers were also proposed [52]. A specific degradation way of the hybrid NGs was noticed; a two-step drop in VPTT appeared, see Figure 5B(I). The degradation of the hybrid NGs was confirmed in the DSC investigations. It is presented in Figure 5B(II). The introduction of the diacryloyl derivative of cystine (BISS) made polymeric PNIPA-based NGs pH responsive and degradable [76]. It was shown, that hybrid NGs containing 3% BISS were highly stable over a wide range of temperature, pH, and ionic strength, see Figure 5C. Furthermore, specific, reversible volume phase transitions were noticed at pH 3.1 for a wide range of temperature that was caused by increased protonation of the carboxylic groups and the decrease in NGs size and the drop in negative charge at the NGs. Deoxyribonucleic acid molecules have been combined with polyethyleneimine (PEI) and covalently bonded with PNIPA to improve transfection efficiency during the hyperthermia treatment [77]. Recently, the design of hybrid NGs where VPTT is marginally higher than the physiological temperature, which promotes drug delivery, was intensively developed [78].

#### 2.1.3. Viscoelasticity

It was mentioned, that due to the high solvation, NGs can behave like solids and liquids. They must possess relatively high viscoelasticity. The main advantage of this property is that NGs’ sizes can be tunable and the gels deformable. In consequence, in internal body capillaries, the NGs can effectively use their viscoelasticity to easily pass through cell barriers. The possibility of control of the viscoelastic parameter in the hybrid NGs depends on: (a) chemical composition of the hybrid NGs, (b) surface charge of solid matter, and (c) crosslinking density of the NGs. The application of biomolecules, as parts of nanogel nets, usually promotes viscoelasticity in the NGs [79]. The studies revealed that the more flexible the NGs, the longer the circulation’s half-life and the lower splenic and lung accumulation seen [80].

#### 2.1.4. Biocompatibility, Degradability, and Reversibility of Volume Phase Transition

The biocompatibility and degradation aspects in the hybrid NGs are especially important from the point of utilization of NGs, as well as controlled release and targeted delivery. The biocompatibility of the hybrid NGs is associated mainly with the presence of high water content and low surface tension [38]. The NGs prepared from known biocompatible polymers or biomacromolecules possess usually little tendency for stimulating the adverse biological responses [81]. The biocompatibility of several nanogel formulations was effectively confirmed by in vitro investigations with the application of cytotoxic assays and the uptake studies [81,82]. However, it should be stressed, that the problem of degradability of the NGs and the utilization of the products of their degradation are not deeply studied in vivo. Recently, the non-toxicity of the nanogel constituents, after the degradation process, was confirmed by in vivo studies for the glutathione (GSH)-responsive hybrid NGs combined from tetralysine and oligoethylenimine [83].

The important ability that NGs possess is the reversibility of the volume phase transition process. This effect is especially helpful for the controlled release of accumulated small molecules/drugs in the NGs. The volume phase transition is directly related to the change in the density of the polymer network. This phenomenon is reversible and is discrete. It was shown, that the way of the introduction of biomolecules into the nanogel network, e.g., accumulation inside the NGs nets and grafting on the external surface of the NGs, can determine the rate and the reversibility of the volume phase transition process [50,51,52,84].

The control of the density of biomolecules introduced as the crosslinkers to the NGs allows precise control of the rate and the reversibility of the shrinking and swelling processes. Moreover, the natural ability of biomolecules to undergo reversible conformational changes, in the presence of various environmental factors, allows the achievement of selectiveness of drug binding and prolonged release of drugs [50,51]. It can be concluded that: (a) the way of the introduction of particular biomolecules and (b) its spatial organization and conformational activity can influence the acceleration or deceleration of volume phase transition in the hybrid NGs. This effect is naturally related to the changes in weak intramolecular interactions occurring in the hybrid NGs, including electrostatic, steric, and specific interactions with the environment.

There are several methods of implementation of controlled degradability based on the use of biomolecules in hybrid NGs. In general, the covalently cross-linked degradable Nanogels are formed during free radical polymerization with labile crosslinkers which allow degradation under specific conditions [85,86]. Other methods applied for the construction of degradable hybrid NGs involve: click chemistry, Schiff’s reaction, thiol–disulfide exchange reaction, and photoreactions [87]. Also, hybrid NGs that are prepared by self-assembling can effectively be degraded due to their weak bonding [88]. Depending on the user’s needs, the biomolecules bioconjugated/grafted to the nanogel surfaces usually need functionalization/modification, at the one end, with labile, cleavable bonds that can be selectively disrupted under given environmental conditions [38,86,87,88]. Such selectively modified biomolecules can be released in a controlled manner. Another way is to introduce biodegradable crosslinking agents inside the biomolecules, e.g., in the middle of the oligonucleotide chain [52]. Then, the biomolecule can act as the hybrid nanogel crosslinker. In general, the polymeric and biomolecule components possessing such functional groups as ester, amine, anhydride, phosphazene, and phosphate esters can introduce degradability into the nanogel nets, see Table 2. Recently it was shown that the modulation of oligonucleotide conformation and the dissociation of the hydrogen bonds can effectively support the degradation of the hybrid NGs [50,51,52].

Finally, hybrid NGs can be degraded by several environmental factors and combinations of them. These factors, as mentioned earlier, are: pH, temperature, solubility, hydrolysis, ionic exchange, charge, enzymatic mechanisms, redox reactions, and photochemical reactions [6]. The NGs that are most frequently used are those temperature and pH responsive. However, the change in NGs related to thermo and pH response is discrete, thus, an uncontrolled burst release of the drug can take place. There is a trend to design multi-responsive NGs to minimize the burst effect and to make the release of the drug both effective and controlled. Very promising is the design of photo-responsive NGs due to the: (a) specific NGs’ preparation method which is very sterile and avoids chemical contamination and (b) short-time drug release, e.g., pulsatile and localized during hyperthermia treatment. This type of stimuli responsiveness is also combined with other kinds of environmental NG responses. Another promising trend is the use of controlled, on-demand NG charge changes for overcoming drug resistance mechanisms.

An overview of the hybrid NGs that could be degraded with particular environmental stimuli is given in the next subsections.

##### Degradation of NGs at Acidic pH

The differences in pH existing inside the body may form the basis for the formation of drug carriers sensitive to pH. Local changes in acidity may generate the response of specific chemical groups introduced into the nanogel network and this may result in relaxation of the polymer chains and release of the drug. The physiological pH value in the healthy tissues and blood is 7.4 while in the malignant tissues it drops to a value between 5.0 and 6.0. The introduction of biomolecules to the polymer network can add sensitiveness to the acidic environment and can promote the degradation of hybrid NGs at reduced pH in the vicinity of the tumor. Various pH-sensitive linkers attached to the biomolecules and the polymer nets, such as acetals, ketals, esters, imines, hydrazones, and vinyl ethers have been used [6].

The acetyl group for the crosslinking of hybrid microgels was used by Bulmus et al. [89]. They prepared acidic precursors—polypeptides functionalized with divinylen. Then, using the inversion emulsion polymerization method, they synthesized microgels crosslinked with acetyl groups based on diacrylate. Next, they found that the microenvironmental polarization of the crosslinking structure can affect the rate of hydrolysis of the introduced linker. The obtained nanostructures were stable at neutral pH and were completely hydrolyzed at an acidic pH in less than one hour. These microgels were tested with Rhodamine B labeled dextran and BSA protein. The release of the drugs was controlled by pH and correspondingly by the degree of degradation of the nanostructure.

Another type of hybrid nanogel containing benzoacetal groups as the degradable linkers was used by Steinhilber et al. [90]. They produced biodegradable NGs based on dendritic polyglycerol by using the inverse precipitation polymerization. It was done without surfactants and CuAAc was used as the reaction initiator. Benzoacetal bonds that were formed in the linking process showed high stability under physiological pH conditions but were cleaved under acidic conditions (pH 5.0). The cleaving was completed in 11 h. Asparginase, a macromolecular protein, has been placed in polymer nanogel networks to test its release during the degradation of the nanogel. At acidic pH, the total release of the protein took place without causing a loss in its enzymatic activity. The atomic force spectroscopy (AFM) was applied to study the degradation process. It was shown that the NGs kept their original shape and size at pH 9.0 due to the presence of acetal linkages, while at lower pH, 4.0, the hydrolysis of bonds and erosion of the molecules occurred.

Berkland et al. [91] synthesized NGs sensitive to acidic environment. Poly-*N*-vinylformamide (PNVF) crosslinked with ketal-based linker was synthesized by reverse emulsion polymerization. The efficiency of encapsulation of lysozyme was tested; it depended on the molar ratio of the monomer to the crosslinking agent. This ratio also affected the half-life of the NGs in an acidic environment. At the ratio of monomer to linker equal to 7:1, the lifetime of the NGs in an acidic medium (pH 5.8) was 90 min. In neutral environment (pH 7.4), it equaled 57 h.

Narain et al. [92] synthesized degradable thermal- and pH-sensitive cationic NGs to deliver genetic material. The NGs consisted of a cationic thermo-sensitive core that allowed easy complexation of negatively charged DNA. The coating was hydrophilic. The release of DNA in the endosomal environment confirmed the degradation of the nanogel structure. Due to the low toxicity and high expression of genes at the target site of this type, core-shell nanoparticles can be potential carriers of genes.

Another type of pH-sensitive moiety used in the synthesis of hybrid NGs is that containing the ester bonds. Matyjaszewski et al. [93] synthesized biodegradable hydrogels based on hyaluronic acid (HA) with a hydrolytically labile crosslinker, bound by the ester bond, and the embedded nanoparticles of 2-hydroxyethyl methacrylate copolymerized with methylacrylate (MA-co-PHEMA). They used the free radical transfer polymerization (ATRP) and the Michael-type addition reactions in the synthesis. The polyester that was formed in the reaction was found to be able to degrade under physiological conditions; the ester bond was degraded. The NGs decomposed into a polymeric sol that allowed the controlled release of fluorescently labeled biomolecules such as isothiocyanate, dextran, and rhodamine B. This biodegradable scaffold of HA with nanoparticles can serve as a potential matrix for encapsulating protein cells for both tissue culture and drug delivery.

Smedt et al. [94] also used ester linkers to synthesize biodegradable cationic microgels by copolymerizing deoxy-hydroxyethyl dexylate (dex-HEMA) with dimethylaminoethyl methacrylate (DMAEMA). The synthesized microgels were used to accumulate siRNA. The genetic material was then released from the microgels at physiological pH (7.4) by hydrolysis of the carbonate esters. It was found that the density of cross-linking may affect the degradation rate of the microgels; in this way, the rate and profiles of siRNA release were regulated. Due to the convenient kinetics of this type of degradation, the nanoparticles can be potential carriers for gene-controlled silencing.

Another type of sensitive pH switch is related to hydrazone linkers which can be hydrolyzed at low pH. It was shown that the degradation of the hydrazone bonds was more than 10 times faster at pH 5.5, compared to pH 7.4. The hydrazone linker was used to synthesize degradable acidic linear polymers, crosslinked nanoparticles, and drugs coupled with the polymer. Hoare et al. [95] have synthesized hydrogels from *N*-isopropylacrylamide and carbohydrate polymers (HA, carboxymethylcellulose, dextran) crosslinked with hydrazine. It was proved that with increasing concentration of the hydrogen ions, the degree of degradation increased. It was confirmed that the hydrazone bond hydrolysis was the major force driving the degradation of the hydrogel.

Ossipov et al. [96] designed a nanogel based on HA and vinyl alcohol. They used the thiol–disulfide exchange reaction and the carbazone chemistry. Hyaluronic acid and doxorubicin (DOX) were bonded to the backbone of polyvinyl alcohol (PVA). It has been shown, that the Dox release process was realized by the cleavage of the carbazole acid bond and was more efficient at acidic pH compared to neutral pH.

Zhang et al. [68] constructed the pH-sensitive cisplatin (CDDP)-crosslinked HA NGs (CDDPHANG) for effective treatment of osteosarcoma, a bone malignance. The obtained NGs possessed prolonged circulation times, effectively preserved the active form of DOX, allowed enhanced intratumoral accumulation, had improved antitumor efficacy, and caused diminished side effects. In another work, they proposed a pH responsive calcium carbonate (CaCO_3_)-crosslinked HA NG for osteosarcoma treatment. The NGs were prepared via a “green” process. Doxorubicin was effectively delivered to cancer tissues and rapidly released in a tumor acidic environment. Novel NGs revealed a high value of suppressed tumor growth, 84.6%.

##### NGs Degradable under the Influence of Enzymes

A new strategy developed by scientists is the synthesis of nanomaterials that decompose under the influence of enzymes present in the physiological environment. Thanks to this type of approach, a drug is released locally at the target site where the concentration of enzyme is the highest.

Wang et al. [97] synthesized biodegradable NGs based on block copolymers (PEG and PEEP) containing polyphosphines. On the surface of the NGs, the lactose moieties have been attached; they can specifically bind to human liver cancer cell line (HepG2) cells of liver cancer. The nanogel was charged with an antitumor drug, DOX. The drug accumulation efficiency was 51%. The release of the drug was generated by employing an enzyme, phosphodiesterase, which catalyzed the breakdown of phosphoesthetic bonds and led to the degradation of the nanogel. It was also shown that due to the ability to direct the lactose-modified nanoparticles to HepG2 cells, the effectiveness of cytotoxicity of DOX-loaded nanoparticles was significantly improved compared to nanoparticles without lactose moieties.

In another paper, Wang et al. [98] showed a similar approach to the synthesis of NGs containing phosphoester moieties coated by polyethylene glycol (PEG) modified by mannose ligands. Nanogels were used for targeted delivery of Vancomycin, an antibiotic. The drug was released after the degradation process performed by the enzymes: phosphatase and phospholipase. In the presence of bacteria, a rapid release of the drug and the inhibition of *Staphylococcus aureus* (MRSA) growth was observed. Mannose-modified nanogel, loaded with the antibiotic, was effectively delivered by macrophages to the site of bacterial infection in vivo. Then, in the presence of bacterial enzymes, the drug was released, which improved the therapeutic efficacy of vancomycin on the infected embryo model of the zebra.

Another approach to the use of enzymes as nanogel degrading agents was proposed by Jiang et al. [99]. They synthesized small, spherical, negatively charged and degradable NGs based on methacrylate HA crosslinked with diethylene glycol diacrylate (DEGDA). Nanogels were degraded by hyaluronidase and lipase enzymes present under physiological conditions. As a result, the targeted delivery of DOX to cancer cells was possible, resulting in increased cytotoxicity of liver cancer H22 cells on a mouse model.

##### NGs Degradable under the Influence of a Redox Substance

The most commonly used reducer in a physiological environment is GSH. It was found that the concentration of GSH is higher in cancer cells than in healthy cells, which may be used for rapid degradation of appropriate nanogel networks and correspondingly higher release of drug substance. The most favored solution is the introduction of the linkers containing SS groups in the structure of the nanogel. These groups undergo a redox reaction under the influence of GSH. Crosslinked by SS bonds compounds are highly stable during circulation in the blood, but under intracellular reducing conditions, they are cleaved, which leads to the degradation and the release of the drug at the target site [100,101].

Matyjaszewski et al. [102] were the first who applied the SS bridges for the design of biodegradable NGs. They synthesized hybrid NGs based on dimethylacrylate (DMA) crosslinked with SS with use of the reverse microemulsion method. In this work, the GSH-mediated release of rhodamine B and the anticancer drug DOX in the NGs was described.

Thayumanavan et al. [103] synthesized the NGs consisting of oligoethylene glycol (OEG) with activated pyridyl disulfides (PDS). The stability of the NGs was studied after the introduction of hydrophobic drugs. The increased release of the drugs, in the presence of GSH, by the degradation mechanism of the NGs was proved.

The new direction observed in the literature is the replacement of the SS moieties with the selenium-based moieties. The selenium groups (Se–Se) are excellent candidates for the dissociation in the presence of oxidizing and reducing agents. Thanks to this fact, it is possible to easily degrade the gel and release the therapeutic substances under the influence of a compound that undergoes redox reactions with Se–Se groups (e.g., H_2_O_2_ and GSH). So far, the synthesis of self-assembled NGs composed of hydrophilic PEG and water-insoluble Se–Se bridges has been proposed. The selenium groups were stable under physiological conditions; they were degraded only under the influence of oxidizing and reducing agents (H_2_O_2_, GSH) [104].

Chen et al. [67] proposed the redox-responsive, dual-transformable, shell-stacked (SNP) NGs for effective treatment of A549 lung carcinoma. Shell-stacked NGs effectively internalized into the xenografted A549 lung carcinoma circa four times deeper compared to the non-transformable one. The constructed NGs possessed prolonged circulation time and the ability to switch negative surface charge to a positive one for efficient penetration and retention in the interstitial space throughout the tumor tissue. They were also pH responsive. Doxorubicin-loaded SNP (SNP/DOX) showed a satisfying antitumor efficacy.

In another two works, the smart redox responsive, SS-crosslinked, cationic poly(l-lysine)–poly(l-phenylalanine-co-l-cystine) (PLL–P(LP-co-LC)) NGs were proposed for bladder cancer treatment with 10-hydroxycamptothecin (HCPT) [71,72]. A prolonged retention period of NGs and redox responsivity resulted in targeted and rapid release of HCPT. The NGs also showed high drug-loading efficiency and an improved tissue penetration capability. The positively charged loading NGs exhibited significantly enhanced antitumor efficacy and reduced side effects.

Feng et al. [73] synthetized GSH-responsive, SS-crosslinked methoxy poly(ethylene glycol)–poly(L-phenylalanine-co-l-cystine) (mPEG–P(LP-co-LC)) NGs for rheumatoid arthritis (RA) treatment and methotrexate (MTX)-targeted delivery. The proposed NGs exhibited efficient internalization, high cytotoxicity, and selective biodistribution in the inflammatory joints of a collagen-induced arthritis mouse model.

Shi et al. [74] showed the potential of dual-responsive (pH and reduction) polypeptide NGs for effective cancer treatment. The selected anticancer drug, DOX, was loaded to the NGs with a very high loading efficiency (96.7%). Then, the efficient cell uptake of DOX was released in a dual environmentally triggered process and caused high cell-proliferation inhibition in vitro. The authors also showed an excellent safety in vivo of the novel hybrid NGs and a great potential for on-demand intracellular delivery of antitumor drugs.

##### NGs Degradable under the Influence of Photochemical Reaction

Photochemical reactions can be easily controlled by applying an appropriate beam of light that initiates the process of nanogel degradation. The substrate molecules can absorb electromagnetic radiation only of the specific energy needed to excite them. The most commonly used photocleavable linker in the NGs is ortho-nitrobenzyl. This linker is widely applied in biomedical applications because it is biocompatible before and after the photodegradation reaction and does not interact specifically with such biomolecules as proteins, DNA, and RNA.

Landfester et al. [105] have synthesized the microgels based on 2-hydroxymethylacrylate (HEMA) and methacrylic acid (MAA) with photosensitive moieties containing o-nitrobenzyl groups by using the reverse microemulsion method. The microgels were sensitive to the pH of the environment as well as degradable under the influence of light. The photolytic degradation experiments were performed using irradiation with UV light. The rate of degradation and changes in the degree of swelling of the microgels were evaluated. It was found that these parameters depended on the pH of the solvent. It was shown that a double stimulation of the NGs by changing pH and light-load enabled effective subsequent release of a model protein—myoglobin. The release mechanisms were a combination of the processes: (a) diffusion controlled, pH-induced slow release and (b) rapid light-induced release through the degradation of the microgels.

Zhao and co-workers [106] constructed another type of degradable NGs sensitive to light. They synthesized thermo-copper and thermoplastic nanoparticles based on self-assembly of coumarin-containing moieties. After the irradiation with a wavelength, λ, of circa 310 nm, either the core or the coating of the nanoparticle was crosslinked. In contrast, the application of the light of a wavelength smaller than 260 nm caused photo-cleaving of the bonds and the degradation of the cross-linked nanogel structures.

### 2.2. Drug Loading Capacity and Controlled Release Aspects

The optimum amount of drug encapsulation is essential for each delivery system and should be carefully considered during the design of a particular drug carrier. The crucial aspects are the optimal loading of the drug for its effective release and the prevention of unwanted drug escape. Typical ways of drug loading into the nanogel nets are: equilibrium partitioning between the solution and the nanogel phases; electrostatic, hydrogen, and hydrophobic interactions; and biodegradable covalent bonding [107]. The introduction into a hydrogel interior network of biological components, such as oligonucleotides, offer an additional option of loading of drugs into the nanogel nets by intercalation of the drug molecules between the parallel planes of the DNA and RNA nucleic bases [50,51,52].

Two main strategies of drug encapsulation in hybrid NGs took place: (a) non-covalent encapsulation in the hybrid polymer nets and (b) conjugation to the polymer chains and biomolecules [108,109]. The introduction of the drug by the first way is done usually during the synthesis step or by loading to synthetized NGs. The encapsulated drug can non-covalently interact with nanogel nets, mainly through electrostatic interactions [110]. A specific way of the non-covalent introduction of the drug into the nanogel nets is the intercalation process between the base pairs of the oligonucleotide biomolecules [50,51,52]. This way is promising from the controlled drug-delivery point, since the balance between several types of drug interaction with the biomolecule and the nanogel can determine the rate of the release process.

The covalent conjugation of the drug molecules to the NGs involves the application of labile bonds for effective release of the drug on the target site, see Table 2. It should be stressed, that stable conjugation and self-assembly of the drug in the nanogel nets depend on the selection of polymeric and biomolecules substrates. The mechanism of drug release previously encapsulated/attached to the hybrid nanogel network is more deeply discussed in Section 4.

### 2.3. Targeted Delivery

The presence of biomolecules in NGs offers enormous potential for targeted delivery of hosted drugs from hybrid NGs to the action place. Another important feature of the application of biomolecules is the use of hybrid NGs as markers for internal imaging, e.g., tumor imaging. Many of the biomolecules attached/encapsulated in NGs behave as high-affinity ligands. Recently, scientists are looking for methods that are aimed at the delivery of a drug to a single cell without affecting healthy ones. The goal is to minimize side effects and increase the effectiveness of the treatment. The active transport of the drug or drug carrier to the site of interaction with the tumor tissue can take place using a mechanism of specific ligand binding to receptors, i.e., antibodies to antigens, and aptamers to transmembrane proteins [19,111].

It was shown that, as the size and charge of the NGs is important from the point of effective internalization of the NGs to the cells, the surface functionalization can significantly raise the cellular uptake of the NGs and its effective delivery to the place of action. This effect is related to the targeted interaction of uniquely and spatially organized biomolecules with cell receptors [19]. Moreover, the ability of small molecules to selectively interact with biomolecules in hybrid NGs promotes the protection of the active form of the drugs. On the contrary, the interactions of the molecule drugs with external molecules/proteins in the body fluids are reduced. Several examples of the hybrid NGs designed for the targeted delivery are given in the following text.

The increased specificity of polymeric nanocarriers has been obtained by binding additional moieties such as folic acid (FA). Folic acid is one of the natural compounds able to interact specifically with folic acid receptor (FR) cells that are highly overexpressed in various types of cancer. The advantage of these structures is the selective deposition and release of the therapeutic substance in the target tissues. A simple strategy for the use of FA, to form target NGs, was proposed by coupling FA with a pullulan hydrogel core through ester bonds. It was proved that due to the increased degree of modification of pullulan-based NGs by FA, a prolonged release of the desired doses of DOX took place in the cancer tissue [112].

There were also carriers based on natural glycosaminoglycan—HA being the main component of the extracellular matrix. It was found that hyaluronan salt shows high affinity for CD44 receptors that are highly overexpressed in cancer cells. An important aspect is that HA undergoes enzymatic degradation with the help of enzymes called hyaluronidases, which makes it possible to claim that the polymeric carriers containing this ligand are biocompatible and biodegradable. The actively directed nanogel was the subject of research by Wooram et al. [113]. Nanogels based on acetylated HA with low molecular weight were synthesized. The properties of the targeted therapy of this type of systems were analyzed using the DOX drug and the degree of interaction of nanosystems on CD44 receptors of the Hela cell line, and the non-binding receptors (Vero) was studied. The charged nanoparticles showed selective cytotoxicity towards the cells with the receptors CD44 binding HA, while they did not act in a control assay with non-binding receptors.

The latest research in the field of obtaining polymer nanosystems is focused on the targeted delivery of medicinal substances with targeted agents—aptamers. Aptamers are a promising tool for combatting viral, cancer, autoimmune, and cardiovascular diseases. Aptamers interact selectively with membrane protein receptors with increased expression in tumor cells. This is based on the spatial self-organization of the aptamer and the interaction with a particular membrane receptor. Nuclear aptamers can recognize various macromolecular compounds, e.g., enzymes, regulatory proteins, growth factors, mono- and polyclonal antibodies, antibiotics and nucleotides. An additional advantage of using aptamers is that they do not show negative immune responses [114].

A pioneering work presenting the use of polymer-modified nanoparticles with aptamers was released 10 years ago. Farokhazadet et al. [115] described the synthesis of PMSA aptamer conjugates with a copolymer of lactic acid and glycolic acid (PLGA). In this model, the aptamer PSMA/drug conjugate was used to control the accumulation and release of the drug, while the PLGA polymer possessed a protective function. After drug accumulation, docetaxel (DTXL) was selected as the agent selectively interacting with the aptamer bound to the cells. It was shown that the nanosystems caused reduced systemic toxicity and significant tumor reduction.

In the following years, modification of the protein mucin 1 (MUC1) aptamer was highly popular due to the high level of overexpression of this protein in many types of tumors, e.g., adenoma. It has been attached to PLGA nanoparticles. The antitumor drug paclitaxel (PTX) was introduced into the system. It was found, that PLGA nanoparticles with the attached aptamer were characterized by enhanced drug delivery in tumor cells with overexpressed MUC1 protein [116].

## 3. Protein and Oligonucleotide-Based Nanogel Hybrids

**Figure 6:**
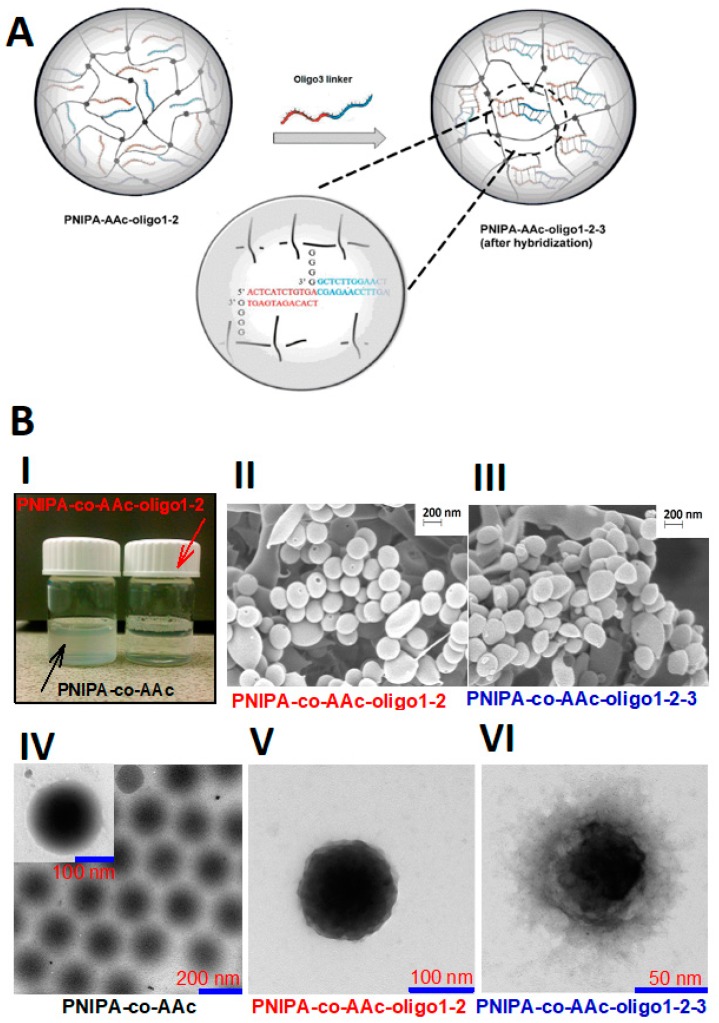
(**A**) Synthesis scheme of poly (*N*-isopropylacrylamide-and-acrylic acid (PNIPA-AAc) -based NGs containing the tri-segment oligonucleotide structure (oligo1-2-3). (**B**) Visualization of hybrid NGs containing the oligonucleotide structures. Comparison of look of PNIPA-co-AAc nanogel solution (left) and PNIPA-co-AAc NGs with immobilized oligo1 and oligo2 strands (right), (**I**). SEM micrographs of typical PNIPA-co-AAc-oligo1-2 NGs (**II**) and PNIPA-AAc-oligo1-2-3 NPs (**III**). TEM micrographs of PNIPA-co-AAc NPs (**IV**), PNIPA-co-AAc-oligo1-2- (**V**), and PNIPA-co-AAc-oligo1-2-3 NGs (**VI**). Reprinted from Reference [51] with permission from the Royal Society of Chemistry.

**Figure 7:**
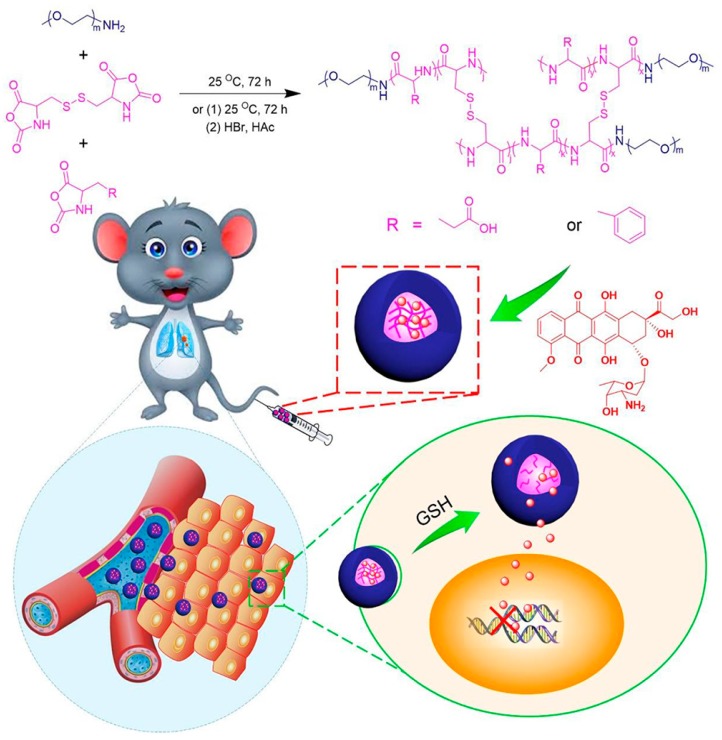
(Top) Synthesis pathways of the reduction-responsive methoxy poly(ethylene glycol)–poly(L-phenylalanine-*co*-L-cystine), (mPEG–P(LP-co-LC))- and methoxy poly(ethylene glycol)–poly(L-glutamic acid-co-L-cystine), (mPEG–P(LG-co-LC)) NGs. (Center) DOX encapsulation to hybrid NGs. (Bottom) DOX-hybrid anogels circulation, intratumoral accumulation, endocytosis, and targeting release after intravenous injection. Reprinted from Reference [117].

Targeted drug delivery is currently one of the most visible areas of research carried out by scientists around the world. Most commonly applied biomolecules for the design of degradable hybrids nanogel are proteins and oligonucleotides, including aptamers. Example hybrid NGs modified by oligonucleotides and proteins are presented in Figure 6 and Figure 7, respectively.

In general, specific ways of introducing biomolecules to NGs determine the structure and functionality of the hybrid NGs. For example, the introduction of programmed tri-segment oligonucleotide structures to PNIPA-AAc NGs resulted in optical clarity of the NGs’ solution and specific core-shell structures, see Figure 6 [51]. Due to the enhanced shrinking of the NGs, the introduced tri-segment oligonucleotide hybrids formed an external NG surface; it was important from the point of view of their interaction with the cells. The specific peptide constituent-based synthesis of red-ox responsive, degradable hybrid NGs led to significant reduction of DOX side effects and effective internalization of the hybrid NGs to the cancer cells, see Figure 7 [117]. An overview of protein- and oligonucleotide-based hybrid NGs is presented in next subsections.

### 3.1. Polymer-Based Background

Hybrid NGs can be prepared from natural or synthetic polymer constituents. The typical synthetic route is the combination of several monomers to design the nanogel base. The most often applied natural polymers used for the synthesis of NGs are: protein polymers (collagen, albumin, fibrin), poly(hyaluronic) acid (pHA), heparin, agarose, polysaccharide polymers (chitosan), chondroitin sulfate, and alginate. The synthetic polymers applied for the construction of NGs are: polyacrylates, polymethacrylates, PLA, PLGA copolymers, PEG, poly(e-caprolactone, PCL), PEI, polyamidoamines (PAMAM), pullulan, and polylysines [118,119].

Typically, the base of a nanogel network is a pH and/or temperature responsive polymer. The group of polymers applied for the design of stimuli responsive NGs include PNIPA, poly(*N*,*N*-diethyl acrylamide), poly(*N*-2-(diethylaminoethyl acrylamide), poly(oligo(ethylene glycol))methacrylate, poly(*N*,*N*-diethylaminoethyl) methacrylate, poly(propylene oxide), poly(*N*,*N*-dimethylamino ethyl) methacrylate, and *N*-vinyl caprolactam [120,121].

### 3.2. Aptamer, Oligonucleotidem, and Nanogel Cooperation

Nucleic acid aptamers appeared as alternative signaling molecules and the objects delivering the drug to the target site. They are single-stranded short fragments of oligonucleotides (DNA or RNA) that, by forming the unique tertiary structures, combine with target molecules of proteins, enzymes, growth factors, antibodies, and even some metal ions. Due to their specific interactions, they are similar to antibodies, but due to their specific structure, they show some differences, which appear to be advantages in their application as the target molecules in targeted therapies. Important advantages of the aptamers are: the lack of immunotoxicity, small size, and, above all, the simplicity of their preparation. The most commonly used method of obtaining aptamers with specific properties is the systematic evolution of ligands by exponential enrichment (SELEX) method. It involves the search and identification of the oligonucleotide libraries to find those with expected characteristics [122].

The disadvantage of applying aptamers is their relatively short half-life under physiological conditions. Therefore, to optimally use them in therapy, they are modified using various molecules or nanostructures. These modifications can be easily carried out by covalent attachment of them to a given type of support, or non-covalent bonding through electrostatic interactions. It should be emphasized that these manipulations do not cause a loss of their original properties, thanks to which we can create new molecular machines for recognizing specific tumor markers useful in modern cancer therapy [123].

Generally, two strategies can be applied for modification of NGs with oligonucleotides and aptamers: (a) design of nanogel spatial networks with either linear or branched DNA-based copolymers during the polymerization process and (b) grafting of obtained NGs. Example groups for chemical modification of aptamers for functionalization of nanogel networks are: amino- and thiol groups, and methyl bridges. The alkylation and dimerization are also used [124]. These modifications improve the affinity of the ligand for the molecule, increase the number of sites binding the target molecules, and above all, increase the resistance of aptamers to attacks by exonuclide enzymes. Table 3 presents a list of the most commonly applied aptamers grafted to NGs for targeted delivery and treatment.

Aptamers can be combined directly with the drugs to form the so-called aptamer–drug conjugates. However, their stability and resistance to the enzymes are somewhat limited. A better solution is the use of NGs combined with aptamers that protect the structure directly and give them some new properties. Explicitly, the formation of the aptamer–ligand complex may introduce a new stimuli-responsiveness of the hydrogels.

Non-covalent connections usually utilize the ability of anthracycline anticancer drugs to intercalate between the base pairs and the specific affinity for the GC or CG pairs in dsDNA. One of the first non-covalent aptamer and drug combinations was the attachment of the PMSA aptamer (prostate cancer) to DOX, which resulted in increased treatment efficacy over the pure drug [125]. That work was followed by many tests that were carried out to modify aptamers with other drug-intercalation sites to increase the capacity of accumulation of the drugs while maintaining the ability to recognize target molecules.

Many non-covalent links of DOX with various types of aptamers such as human epidermal growth factor receptor 2 (HER2)–DOX (breast cancer therapy) or MUC1–DOX (lung cancer therapy) were reported in the literature. All of these conjugates demonstrated good cellular absorption and targeted delivery, as well as reduced cytotoxicity in the healthy cells, as compared to unbound DOX [126,127]. Despite the benefits of using these types of conjugates, these connections are still not perfect. The disadvantages of such systems are primarily the instability of the conjugate in the physiological environment, the short half-life in vivo associated with their low molecular weight and the low efficiency of packing of the drug in the aptamer body.

The second way to create conjugates of aptamers is to attach them with covalent bonds. The precursors in the covalent conjugation of the sgc8 aptamer with tyrosine kinases, PTK7, and DOX were Huang et al. [137]. The substrates of the conjugate were connected using a hydrazone bond. The bond underwent the degradation in an acidic environment. This allowed the release of the drug in the environment of the tumor cells. The conjugate was tested in vitro on a cell line of human lymphoblastic leukemia. Wang et al. [138] proposed a modular synthesis of sgc8 aptamer conjugates with a drug in which a substantial amount of the drug can be specifically coupled to each aptamer molecule. For this purpose, phosphoramidite was synthesized as a carrier of 5-fluorouracil (5-FU)—a drug used in tumors of the large intestine and pancreas. A photocleaving linker (PC) was also used to release the drug under the influence of light. The carrier was then coupled to the aptamer which resulted in a high drug accumulation performance. The cellular studies carried out on colon cancer cells using this conjugate demonstrated the suppression of tumor cell proliferation.

In a recent study, Zhu et al. [114] used a reaction to design conjugates containing multiple copies of the drug molecules attached to one aptamer. They were inspired by the fact that anthracycline drugs (DOX, epirubicin) and cis-diamminedichloridoplatinum(II) (CDDP) inhibit the proliferation of cancer cells by creating the adducts with DNA to induce the cell apoptosis.

The aptamer–drug conjugate was formed using formaldehyde as a crosslinking agent to form a methylene linker between the 3-NH_2_ group of DOX and 2-NH_2_ deoxyguanosine (dG). Using the sgc8 aptamer and the AS1411 antinuclear aptamer, it was proved that the synthesized conjugates inhibited tumor growth in leukemia and liver cancer.

Farokhzad and colleagues [139] prepared a triblock copolymer (TCP) combining PLGA, PEG, and aptamer A10 (Apt). This type of aptamer conjugate with polymer nanoparticles has been tested to target prostate cancer cells. It was found that the high density of aptamer on NGs’ surface increased the accumulation of nanoparticles in such organs as the liver and spleen. Therefore, it was necessary to introduce an aptamer targeting the tumor in a balanced way. By using different proportions of the aptamer to the PLGA–PEG copolymer, a suitable concentration of the surface aptamer relative to the polymer was found. The maximum absorption of the NGs into the cells of the prostate cancer was determined in vitro and in vivo.

Another type of approach to immobilization of aptamers is the formation of polymeric nanoparticles combined with polymeric micelles. Tan and colleagues [140] presented this approach in their work. Hybrid PLGA nanoparticles with a hydrophilic lipid–polymer envelope were synthesized by nanoprecipitation and self-assembly of lipid micelles on the polymer core. The nanosystem shell was composed of DSPE–PEG lecithin and a PEG–aptamer lipid with hydrophilic properties. After self-organization, two types of drugs were loaded into this type of media: (a) a hydrophobic PTX-drug, which accumulated on the hydrophobic PLGA core and (b) the hydrophilic DOX in the lipid-modified shell. Doxorubicin, DOX was added using the intercalation process between the base pairs in the double-stranded aptamer structure. These types of nanosystems have been tested for the co-delivery of two types of drugs to the cancer cells of human lymphoblastic leukemia. The cytotoxicity results of this type of co-delivery systems have shown selectively the successful drug delivery and increased anti-cancer efficacy.

Another recent work demonstrates the use of block copolymers of polyethylene glycol, lactic acid, and glycolic acid in attaching the anti-nucleation aptamer AS1411 [141]. The obtained nanoparticle and aptamer conjugates were combined with the gemcitabine drug. The interaction of nucleolin 549 target cell receptor with the obtained conjugate was tested in vitro by flow cytometry and fluorescence microscopy. The cytotoxicity was also tested in Chinese hamster ovary cancer cells in the presence and absence of nucleolin. It was shown that the release of the drug is high in the cells targeted by aptamer-functionalized nanoparticles compared to the nanoparticles without aptamer.

Oligonucleotides can be successfully used in the construction of an effective drug reservoir system and for the controlled and targeted release of drugs by the hybrid NGs. The hybridization of DNA strands was also employed in the nanogel construction. The presence of double-stranded DNA and its ability to undergo reversible conformational changes in response to temperature gives a potential for reversible modulation of phase transition of the hydrogel lattices and the achievement of selectiveness of drug binding and prolonged release of drugs. Liwinska et al. [51] designed a specific versatile network, proposed a biocompatible hybrid nanogel where two DNA strands were covalently attached to the polymer net and then hybridized with the third 50/50 complementary oligonucleotide. It was demonstrated that the DOX drug can be effectively stored (by intercalation) in DNA hybrids and then slowly released from the novel NGs under physiological and hyperthermic conditions. This method of Dox accumulation (intercalation) allowed preserving the activity of the drug. The mechanism of the Dox release from the NGs at selected temperatures was a result of two reversible processes: (a) the oligo hybrid conformational change and (b) the phase transition of the hydrogel. The proposed NGs worked well in vitro experiments with Hela and Insulinoma cancer cells.

The hybridization process was also used in the surfactant- and initiator-free polymerization process of NGs containing appropriately sequenced DNA molecules as the crosslinkers, to synthesize nano-sized and degradable NGs [50]. Three-segment oligonucleotide hybrids were introduced as the crosslinkers to the PNIPA-AAc nanonetwork. Several processes, including: (a) binding of the drug through intercalation, (b) change of conformation of the DNA hybrid crosslinkers, and (c) degradation of the crosslinkers, gives novel possibilities for much more effective and wider controlled release of drugs.

### 3.3. Amino Acids and Proteins in NGs

Proteins and amino acids can be similarly introduced to nanogel networks by (a) non-covalent self-assembly encapsulation and (b) conjugation/grafting to polymer chains. The method of modifying NGs by electrostatic interaction of polymers is relatively simple; however, the control of the degradability of the NGs and their targeted delivery aspects are less convenient compared to covalent conjugation-based methods. The ability to control the spatial organization and hierarchization of the proteins is higher for covalently conjugating proteins. On the other hand, the proteins bound to NGs in a covalent way are stable in biological fluids for a longer time; thus, they can interact with biological fluid constituents [78].

In general, proteins are very fragile biomolecules and the effectiveness of introducing them to the NGs depends on a combination of several environmental factors, such as: the presence of active sides in the proteins, the ability of additional functionalization of proteins with labile groups, and the requirement of mild reaction conditions. Most commonly used natural amino acids presented in proteins for = functionalization with NGs are: lysine and cysteine, where the amino group and SS bonds are used for the formation of the labile bonds. A more convenient way is the previous modification of the protein with a labile linker or ligand. It was shown that such modification does not disturb the protein spatial structure required for its functionality [142]. Such groups as amine-reactive aldehydes, cysteine-reactive maleimides, and pyridyl disulfides are used for conjugation of proteins to NGs [143].

Several types of NGs functionalized by proteins and amino acids were proposed in the literature. Akiyoshi et al. [144] presented cholesterol-modified pullulan nanogel (CHP) that was non-covalently bonded by employing the hydrophobic interactions with various proteins. It was shown that the rise in temperature during the thermal denaturation drastically destabilized the proposed protein/nanogel complexes. Polysaccharide-based NGs for the entrapment of proteins were also proposed [29]. In general, it was shown that the stability of NGs non-covalently modified by proteins was affected not only by the denaturation-promoting conditions but also by higher concentrations of the proteins. The raspberry-like assembly of NGs was proposed by the application of the crosslinker based on acrylate-group modified CHP with thiol-modified tetra-armed PEG. This combination allowed the extension of the time of protein/nanogel stability [145].

Nagahama et al. [146] presented the hybrid self-assembled NGs prepared from poly-(L-lactide)-grafted dextran for the release of lysozyme. Thienen et al. [147] obtained the stable lipid-coated dextran NGs for the release of proteins. In another try, Matyjaszewski et al. [148] designed the hybrid NGs based on *N*-(2-hydroxypropyl)methacrylamide and *N*,*N*′-bis(acryloyl)cystamine for the non-covalent encapsulation of proteins with very high loading efficiency under reductive conditions. Disulfide bond crosslinked hybrid NGs for the accumulation of high-weight proteins, e.g., cytochrome C, and their release under reductive conditions were also reported [61]. The poly(vinylformamide)-based NGs for loading of lysozyme and its high efficient release were proposed [149]. Other NGs were applied for entrapment and delivery of vaccines to act as the synthetic adjuvants and for conjugation with immunostimulants [150].

Li et al. [70] presented sarcoma-targeting peptide-decorated SS-crosslinked polypeptide NGs (STP-NG) for enhanced intracellular delivery of shikonin (SHK), and effective osteosarcoma treatment. These sarcoma receptors and redox dual-responsive NGs exhibited excellent potential for inhibiting osteosarcoma progression with minimal systemic toxicity. With effective drug loading, NG cell internalization and intracellular targeted delivery were achieved.

Chen et al. [75] proposed targeted delivery NGs based on both receptor-mediated targeting (RMT) and environment-mediated targeting (EMT). The PMNG NGs, designed with both phenylboronic acid (PBA) and morpholine (MP), were reported for not only RMT, via specific recognition of sialyl (SA) epitopes, but also EMT toward acidic pH. The dual targeting NGs exhibited a great targeting effect of DOX and a high potential for inhibiting the primary tumor growth as well as tumor metastasis on B16F10 melanoma-grafted mouse model.

## 4. Mechanisms of Controlled Drug Release from Hybrid NGs

**Figure 8:**
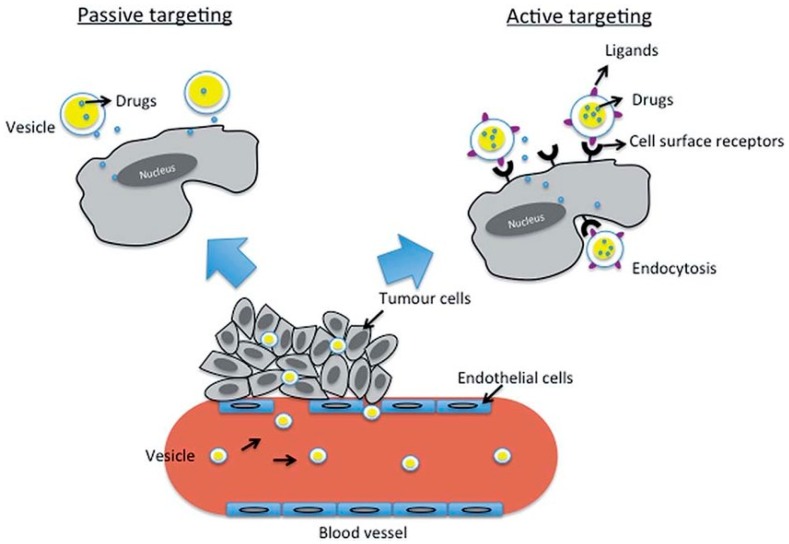
Scheme of passive and active drug delivery from hybrid nanostructures. Reprinted from Reference [153] with permission from the Royal Society of Chemistry.

In general, small substances can be delivered from hybrid NGs in active and passive targeting ways, see Figure 8. Passive targeting is a consequence of accumulation of NGs due to the enhanced permeability and retention (EPR) effect effect that is characteristic for leaky cancer tissues. Strongly hydrophilic NGs promote the accumulation of NGs by passive targeting. Active targeting requires a ligand–cell receptor to enable the penetration of the NGs into the cells.

It was mentioned, that different stimuli can control and trigger the release process of drugs from the NGs. Moreover, more than one release mechanisms may occur in hybrid NGs. For hybrid NGs, the major drug release mechanisms can be realized by:

(a) diffusional transport,
(b) the swelling-shrinking process and degradation, and
(c) presence of chemicals.

### 4.1. Improved Nanogel Permeability and Drug Diffusivity Relevant to Controlled Release

Controlled drug-release systems provide a sustained delivery of drugs which should provide a desired concentration of the drug in cells during the entire treatment cycle. Diffusion-controlled hybrid NGs possess an advantage of the release of the drug in the near zero-order kinetics. Since diffusion is the rate-limiting step, the design of the hybrid NGs can be realized with an attention to the permeability of the drug molecules. Generally, the NGs widen the pore size and enhance the drug diffusion. This is done by (a) swelling process and (b) the degradation process. The acceleration of the diffusion process can be also obtained by changing several other physicochemical factors: temperature, pH, displacement of counterions present in the environment, and external energy sources. The environmental responses stand mainly with decreasing the NGs’ size and increasing drug solubility, thus making the diffusion process faster. On the other hand, typical nano-sized objects possess relatively high surface-to-volume ratio and the drug molecules are rather accumulated on the surface. Then, the distance for the diffusion process is reduced, thus, the inconvenient burst effect can occur. The burst effect is minimalized by increasing the crosslinking density, creating multilayer systems and modifying the surface. A very interesting aspect is to control the diffusion of molecules by changing the spatial organization and conformation of the biomolecules present in the NGs [151,152].

### 4.2. Improved Breaking of Thermo- or Photo-Labile Bonds

The rise in temperature which occurs upon application of remote stimuli can provide sufficient energy to break thermolabile covalent bonds. This is evident in the triggered release of host–guest conjugates NGs. In this type of nanostructures, guest molecules are bind with the host (usually inorganic matrix) by Watson–Crick base pairing which is a type of bonding which is relatively unstable in changing temperature. This is because Watson–Crick base pairing is different from the conventional covalent bonding or hydrogen bonding in such a way that its binding strength is intermediate of that of covalent bonds’ and hydrogen bonds’. As such, local heating generated by the stimuli application would be sufficient to break such bonding. Such host–guest nanostructures are usually used in gene delivery applications. Nanostructures which adopt this bond cleavage mechanism are usually easy to fabricate in terms of the immobilization technique of the desired payloads. Furthermore, such nanostructures usually possess higher loading capacity and a higher degree of control over the release behavior. However, there are a few drawbacks that this type of nano-vehicles may experience. One of them would be the inability of the nanostructures to fully encapsulate and quench the chemotherapeutic payloads, thus raising some concerns over the possibility of an incursion of toxic side effects. Another one would be that bond cleavage often requires a high amount of energy, and thus, a high degree of local heating—this might also induce undesired side effects. Furthermore, there exists a contradictory notion such that to form stable host–guest nanoconjugates, strong covalent bonding would be desirable but in terms of bond cleavage, the opposite would be true. As previously mentioned in Section 3, gating components.

The application of localized thermal and photothermal stimuli can directly control the cleavage of thermolabile covalent bonds. This method of drug release is particularly useful for delivery of directly conjugated drugs and biomolecules to the nanogel surfaces. As well, the release of the drug during the destabilization and spatial reorganization of the Watson–Crick base pairing is very effective [50,51,52]. It was shown, that such a type of drug release is dominating in the drug release from the oligonucleotide/nanogel hybrids [154]. However, a long or uncontrolled heating process can lower the therapeutic efficiency due to the destabilization of the active form of the drug. The protection of the active form of the drug, by for example, intercalation between the base pairs of the double-stranded DNA, is particularly important. Therefore, a balance between the ability of the drug release, the degradability aspect, and the rate of nanogel volume phase transition process are crucial from the point of view of effective release of the drug.

### 4.3. Influence of Phase Transition and Reversible Change of Conformation

The drug release process can be controlled by volume phase transition changes of biomolecule nanogel hybrids. As it was mentioned in Section 2, the phase transition process can be reversible and controlled by several types of environmental stimuli. Thus, there is an opportunity to release the drug in a pulsatile way, even mimicking biological rhythm [50,51,52,103,155]. Importantly, the NGs can be regenerated before loading another portion of the drug. Two processes can occur in hybrid NGs during the drug release: (a) the conformational transition of the NGs and (b) conformational changes/spatial reorganization of the biomolecules introduced to the NGs. The combination of both types of phase transition can introduce a unique possibility of the drug release. This mechanism of drug release can be accelerated/controlled by the introduction of labile bonds and dedicated polymeric moieties for its independent response to particular stimuli. The drug release process is an effect of intensity/amplitude and period of exposure to particular stimuli. Furthermore, to better control the drug release process, the design of hybrid NGs with a VPTT above 37 °C should be considered. It should be ensured that the drug is protected in the nanogel net and the leaking effect or the premature release is strongly limited. Finally, the thermoresponsive hybrid NGs with VPTT of 40–45 °C would be very effective for drug release by the reversible conformation mechanism. As it was mentioned, the introduction of biomolecules into nanogel networks can increase the VPTT temperature of conventional polymer applied for the design of nanogel networks.

## 5. Conclusions and Future Perspectives

Modern medicine is focused, among other things, on finding methods of controlled delivery of therapeutic drugs only to required destinations. In this respect, the combinations of biomolecules and NGs are perspective, since they can simultaneously act as effective reservoirs of medicines and as targeted drug carriers. Binding of biomolecules to NGs allowed scientists to increase the concentration of the molecules-ligands that target the cells molecule, to improve the aspect of drug accumulation, to protect the active form of the drug and, above all, to release the drug in a controlled manner. Various effective combinations of biomolecules and gel nanoparticles have been proposed. The group of applied biomolecules includes proteins, amino acids, nucleic acids, and aptamers. Furthermore, it was shown that many stimuli-responsive biomolecule–nanogel hybrids significantly enhanced the therapeutic effect of successfully increased doses of intracellularly delivered drugs. A very interesting aspect is the mutual influence of bound biomolecules and the NGs on the physicochemical parameters/properties of the hybrids. These properties allow the design of drug carriers with unique possibilities of controlling the release of drugs. Nevertheless, it is clear that more in vivo studies should be conducted to describe and explain the metabolic pathways and the removal of biomolecule-nanogel hybrids. Also, the understanding of the mechanisms of degradability and lability of biomolecule–nanogel conjugates under in vivo conditions should be improved. Finally, new unique methods of binding biomolecules, e.g., as the crosslinkers in NGs, should be deeper explored. The fusions of biomolecules and NGs are promising types of drug carriers. In particular, the aspect of controlled spatial changes of molecules bound in NGs, the correlation with oscillating/pulsating changes in environmental conditions, and reversible behavior of the NGs can contribute to the development of carriers with new mechanisms of drug release and controlled delivery.

## Figures and Tables

**Table 1:** Responsiveness of selected degradable hybrid NGs.

Type of Hybrid Nanogel	Type of Response-Stimulus	Type of Released Drug/Compound	Reference
Chitosan/PEO	pH	Amoxicillin, metronidazole	[58]
Y-gel-aptamer/DNA	redox-GSH	Aptamer	[21]
MUC1 aptamer/PLGA	MUC1 protein, ultrasounds,	Aptamer	[59]
FA/pullulan	Hyaluronic receptors	Doxorubicin, DOX	[37]
Protein/PEGMA-co-PDSMA	redox-GSH	BSA protein	[60]
Protein/PEG-P(HEMA-co-AC)	redox-DTT	Cytochrome C	[61]
BSA protein/chitosan	pH	DOX	[62]
FA/PEO-b-PMA	Folate receptor, pH	CDDP, DOX	[63]
Antibodies vaccine/cholesteryl pullulan	NY-ESO-1 and HER2 antigens	Antibodies	[64]
imidazoquinoline-based TLR7/8/poly(mTEGMA-b-HEMAm)	pH	TLR7/8 agonist	[65]
antibody/PEI/DNA/HA	CD44 receptor, surface charge	Aptamer	[66]
SN NPs with -SS-crosslinkers	redox-GSH, pH	DOX	[67]
CDDP crosslinked-HA	pH	DOX, CDDP	[68]
CaCO_3_-crosslinked HA	pH	DOX	[69]
sarcoma-targeting peptide-SS-crosslinked polypeptide	Sarcoma receptors, redox-GSH	SHK	[70]
SS-crosslinked (PLL–P(LP-co-LC))	redox-GSH	HCPT	[71,72]
SS-crosslinked (mPEG–P(LP-co-LC))	redox-GSH	MTX	[73]
Polypeptide-based	redox-GSH, pH	DOX	[74]
nanogels with PBA and MP	sialyl epitopes receptors, pH	DOX	[75]
Tri-segment oligonucleotide/PNIPA-AAc	pH, temperature, surface charge	DOX	[50]
Oligonucleotide-crosslinked/PNIPA-AAc	pH, temperature, surface charge	DOX	[51]
Oligonucleotide-SS-/PNIPA-AAc	redox-GSH, temperature, pH surface charge	DOX	[52]

PEO—Poly (ethylene oxide), DNA—deoxyribonucleic acid, PLGA—poly (lactic-co-glycolic acid), FA—folic acid, PEGMA—methyl ether methacrylate, PDSMA—pyridyl disulfide methacrylate, PEG—poly (ethylene glycol), HEMA—Poly (2-hydroxyethyl methacrylate), PMA—poly (methyl acrylate), TEGMA—tri (ethylene glycol methacrylate), PEI—poly (ethylenimine), HA—hyaluronic acid, SN NPs—shell stacked nanoparticles, SS—disulfide bonds, CDDP—cis-diamminedichloroplatinum, cisplatin, CaCO_3_—calcium carbonate, PLL–P(LP-co-LC—poly(l-lysine)–poly(l-phenylalanine-co-l-cystine), mPEG–P(LP-co-LC)—methoxy poly(ethylene glycol)–poly(L-phenylalanine-co-L-cystine), PBA—phenylboronic acid, MP—morpholine, PNIPA—poly (*N*-isopropylacrylamide), AA—acrylamide acid, GSH—glutathione, DTT—dithiothreitol, DOX—doxorubicin, SHK—shikonin, HCPT—10-hydroxycamptothecin, MTX—methotrexate, MUC1—protein mucin 1, BSA—Bovine serum albumin, TLR—toll-like receptors, NY-ESO-1—New York esophageal squamous cell carcinoma 1 antigen, HER2 - human epidermal growth factor receptor 2, CD44—cell surface glycoprotein.

**Table 2:** Labile bonds introduced for effective release from hybrid NGs.

Linker Type	Chemical Structure	Degradation Conditions	Ref
Acetalic linker	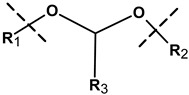	Hydrolysis in acidic medium, pH = 5	[60]
Ketal linker	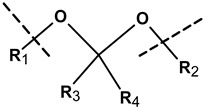	Hydrolysis in acidic medium, pH = 5.5	[61]
Ester linker	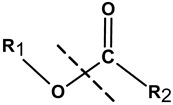	Hydrolysis below physiological pH	[62]
Vinyl ether linker	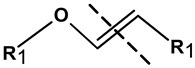	Hydrolysis in acidic medium, pH <5	[63]
Linker based on ortho-nitrobenzyl ester	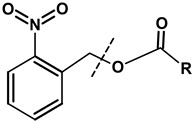	Hydrolysis under the influence of UV 315–390 nm	[64]
Linker based on disulfide or diselenide bridges	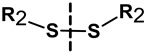 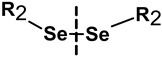	Hydrolysis in the presence of GSHcarboxyethylphosphine tris (TCEP), and Dithiothreitol (DTT)	[65,66,67]
Phosphoester linker	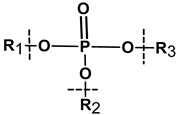	Hydrolysis in the presence of phosphatase or phospholipase enzyme	[68]

**Table 3:** Most commonly applied aptamers for combination with NGs.

Aptamer Type	Cancer Type	Type of Drug	Type of Nanogel	Ref.
RNA/PSMA	Prostate cancer	Docetaxel	PLGA-b-PEG	[128]
RNA/PSMA	Prostate cancer	Doxorubicin	Dendrimers	[129]
RNA/PSMA	Prostate cancer	Docetaxel	PLGA-liposomes	[115]
DNA/AS1411	Pancreatic cancer	Curcumin and gemcitabine	PLGA/magnetic	[130]
DNA/AS1411	Breast, pancreatic cancer	Doxorubicin	pPEGMA-PCL-pPEGMA	[131]
DNA/MUC1	Pancreatic cancer	Paclitaxel	PLGA	[132]
DNA/MUC1	Colon cancer	SN-38	Chitosan	[133]
RNA/EpCAM	Breast cancer	Doxorubicin	PLGA/PEG	[134]
RNA/EpCAM	Breast cancer	Curcumin	PLGA-lecithin-PEG	[135]
PDNA/PGDF-B	Ovarian cancer	PGDF-B	Streptavidin-coated polystyrene, poloxamer	[136]
RNA/Ep	Breast cancer	siRNA	PEI	[136]

RNA—ribonucleic acid, PSMA—prostate-specific membrane antigen aptamer, AS1411—anti-nucleolin aptamer, EpCAM—epithelial cell adhesion molecule aptamer, PDNA—plasmid DNA, PGDF-B—platelet-derived growth factor B, Ep—epithelial cell, SN-38—an antineoplastic drug, the active metabolite of irinotecan drug, PLGA-b-PEG—poly (ethylene glycol) methyl ether-block-poly(lactide-co-glycolide), pPEGMA-PCL-pPEGMA—poly (polyethylene glycol methacrylate)-poly(caprolactone)-poly(polyethylene glycol methacrylate).
